# Genomic and Transcriptomic Variations of *INSR* Are Associated With Dysregulation of Circulatory miRNA‐21 and miRNA‐146a in Type 2 Diabetes Mellitus

**DOI:** 10.1155/jnme/9980111

**Published:** 2026-07-22

**Authors:** Hira Gul, Nosheen Masood

**Affiliations:** ^1^ Molecular Genetics Laboratory, Department of Biotechnology, Fatima Jinnah Women University, Rawalpindi, Pakistan, fjwu.edu.pk

**Keywords:** diabetes, *INSR*, linkage disequilibrium, microRNAs, SNPs

## Abstract

**Background:**

This cross‐sectional case–control study aimed to investigate the association between germline and sporadic variations in the *Insulin Receptor (INSR)* gene, risk factors, and the differential expression of circulating miRNA‐21 and miRNA‐146a in the onset of Type 2 diabetes mellitus (T2DM).

**Method:**

A total of 800 T2DM patients and 600 nondiabetic age‐ and gender‐matched individuals participated in single nucleotide polymorphism (SNPs) screening in the *INSR* gene via polymerase chain reaction (PCR), followed by Sanger sequencing. Transcriptional expression was quantified using reverse transcriptase PCR (RT‐PCR). Relative expression levels of circulating miRNAs were determined by real‐time PCR.

**Results:**

A significant (*p* < 0.05) association of Exon 17 *INSR* SNPs with T2DM, including a single base substitution mutation at Codon 115 (rs1799817; CAC > CAT) and Codon 83 (rs1799816; GGT > GAT), was found. However, a deletion mutation at Codon 186 (rs1052371; delT) showed no significant association with the disease. Linkage disequilibrium analysis (*r* ^2^ > 0.8) indicated a strong correlation among these SNPs and T2DM. *INSR* and miRNA‐21 levels were significantly downregulated in T2DM patients, while circulating miRNA‐146a was upregulated, as shown by relative expression analysis. ROC curve analysis indicated that miRNA‐21 and miRNA‐146a exhibited high diagnostic efficacy for T2DM. Furthermore, combined detection of these miRNAs showed greater potential as biomarkers for insulin resistance and metabolic complications in T2DM, particularly in this study population.

**Conclusion:**

This study highlights a significant association between SNPs in Exon 17 of *INSR* and the differential expression of circulating miRNAs in T2DM.

## 1. Introduction

Diabetes mellitus (DM) is a heterogeneous group of metabolic disorders characterized by persistent hyperglycemia, which arises from defects in insulin secretion, insulin action, or a combination of both. Chronic hyperglycemia in diabetes is associated with progressive damage, dysfunction, and failure of various organs, including the eyes, kidneys, nerves, heart, and blood vessels [1]. The pathogenesis of diabetes involves multiple mechanisms, ranging from autoimmune destruction of pancreatic β‐cells, leading to insulin deficiency, to insulin resistance caused by cellular abnormalities. These disturbances disrupt carbohydrate, fat, and protein metabolism due to deficient insulin activity in target tissues, resulting from either inadequate insulin secretion or impaired tissue responsiveness to insulin. Notably, both impaired insulin secretion and resistance often coexist in individuals with diabetes, making it challenging to identify the primary driver of hyperglycemia [2].

Insulin functions as a ligand, binding to insulin receptors (*INSRs*) on target cells to activate intracellular signaling pathways essential for nutrient homeostasis [3, 4]. The *INSR* is a tetrameric protein composed of two extracellular α subunits and two transmembrane β subunits, linked via disulfide bonds [5–7]. Upon insulin binding, conformational changes in the receptor trigger the activation of various intracellular pathways within seconds, regulating blood glucose levels. Defects in *INSR* signaling are recognized as key molecular mechanisms underlying insulin resistance, with mutations in the *INSR* gene contributing significantly to the pathogenesis of Type 2 DM (T2DM) [8, 9].

MicroRNAs (miRNAs) are critical regulators of gene expression [10–13], including those involved in the insulin signaling pathway [14, 15]. The association of *INSR* Exon 17 with miR‐146a and miR‐21 in diabetes highlights the intricate interplay between genetic variations and miRNA‐mediated regulation in metabolic disorders. *INSR* Exon 17 encodes a crucial region of the *INSR*, and its polymorphisms or altered splicing can influence insulin signaling pathways [16]. miR‐146a and miR‐21, known to regulate inflammation and cellular stress responses, can target key genes within these pathways [17, 18]. Dysregulation of miR‐146a and miR‐21 has been implicated in insulin resistance and beta‐cell dysfunction, core features of diabetes [19–23]. Their interaction with *INSR* Exon 17 could exacerbate metabolic imbalances, providing insights into potential biomarkers and therapeutic targets for diabetes management [21, 22]. However, the role of *INSR* gene mutations and specific miRNAs in the pathogenesis of T2DM has not been extensively studied in Pakistan. Therefore, this study aims to investigate single nucleotide polymorphisms (SNPs), expressional variation in *INSR*, and the expression of miRNAs in relation to T2DM and its complications in Pakistani patients.

## 2. Research Design and Methods

This study was conducted at the Molecular Genetics Laboratory, Department of Biotechnology, Fatima Jinnah Women University, Rawalpindi, Pakistan, following approval by the university’s ethical committee (FJWU/EC/2023/64) and local hospital boards. Participants, including 800 T2DM patients and 600 nondiabetic controls, were recruited from hospitals and diagnostic laboratories in Rawalpindi. Written informed consents were obtained from all participants in compliance with the World Medical Association Code of Ethical Principles for Medical Research (Declaration of Helsinki). Peripheral venous blood samples were collected from participants in EDTA vacutainers under written informed consent. Each sample was assigned to a unique coded identifier at the time of collection. No personal identifiers were included in laboratory analyses. DNA and RNA extraction were performed using coded samples only, and all genetic and transcriptomic data were analyzed without linkage to participant identity. Data were stored on password‐protected institutional systems accessible only to authorized members of the research team. Patients with confirmed T2DM were recruited during routine hospital visits, and clinical and biochemical parameters were obtained from laboratory reports at the time of enrollment. Peripheral blood samples were collected at a single time point for genetic and transcriptomic analyses.

### 2.1. Sample Size Justification and Power Analysis

Post hoc power analysis was performed using G∗Power 3.1.9.7 for sample size estimation. At an alpha level of 0.05, the sample size of 800 cases and 600 controls is sufficient to obtain a statistical power greater than 90%.

Eligibility criteria for T2DM patients adhered to the guidelines of the American Diabetes Association (ADA) and World Health Organization (WHO), which include fasting plasma glucose (FPG) levels > 126 mg/dL (7.0 mmol/L), 2‐h oral glucose tolerance test (2h‐OGTT) results > 200 mg/dL (11.99 mmol/L), or glycated hemoglobin (HbA1c) levels ≥ 6.5% (48 mmol/mol). Patients with conditions such as other types of diabetes, cancer, chronic liver or kidney disease, autoimmune disorders, or endocrine abnormalities were excluded. Data on demographics, disease duration, obesity, cardiovascular history, lifestyle, and smoking habits were collected using a structured questionnaire.

Obesity and T2DM susceptibility were defined as body mass index (BMI) > 25/30 kg/m^2^. Blood pressure measurements (systolic and diastolic) were obtained using a mercury sphygmomanometer, with thresholds of > 140/90 mmHg indicating hypertension. Overnight fasting blood samples were collected for biochemical evaluations, including FPG, HbA1c, low‐density lipoprotein cholesterol (LDL‐c), high‐density lipoprotein cholesterol (HDL‐c), and triglyceride (TG) levels.

Genomic DNA was extracted from EDTA‐anticoagulated blood using the phenol–chloroform method [24, 25]. Polymerase chain reaction (PCR) amplification targeted three key *INSR* variants: rs1799817 (C/T), rs1052371 (T > C), and His1058 (C > T) within Exon 17 of the tyrosine kinase domain. Primers were designed using tools such as Primer3, NCBI Primer‐BLAST, and UCSC Genome Browser, ensuring specificity and compatibility (Table 1) (Supporting 1, 2, and 3).

**TABLE 1:** Primer sequences, mutation, melting temperature, GC content, and PCR product length of *INSR* primer sequences.

INSR variant	Primer sequence	GC %	Product length (BP.)	TM
rs1799817 C > T	F‐CCAAGGATGCTGTGTAGATAAG‐3′	45	317	60.8
	R‐ CCAACAGAGGACTCTTGGTCT‐5′	52		

rs1052371 T/C	F‐CTAGTCAAGGTCCAGAACC‐3′	53	223	58.4
	R‐AGGCACACAAAGGGACGAG‐3′	58		

rs1799816 G > A	F‐ TGGGTGGAAGGTGGCGTCAGA ‐3′	45	317	61.8
	R‐TCAGGAAAGCCAGCCCATGTC‐3′	57		

The PCR reaction consisted of a 50 μL mixture containing 200 ng DNA, 40 pmol of primers, 1.5 mM of MgCl_2_, 0.2 mM of dNTPs, and 1.25 U of Taq polymerase. Thermal cycling included initial denaturation at 94 °C (5 min), 35 cycles of denaturation at 95 °C (30 s), annealing (40 s), extension at 72 °C (45 s), and a final extension at 72 °C (10 min). PCR products were visualized on 1.5% agarose gels stained with ethidium bromide. Sequencing was performed using the Sanger method, and chromatograms were analyzed with Chromas software.

Peripheral blood mononuclear cells (PBMCs) were isolated using Histopaque density gradient centrifugation. Total RNA and miRNAs were extracted using the TRIzol reagent and the PureLink™ miRNA Isolation Kit (K157001), respectively, and quantified using a NanoDrop spectrophotometer. Samples with an A260/280 ratio of 1.9–2.2 and an RNA integrity number (RIN) > 8.0 were used.

First‐strand cDNA synthesis utilized SuperScript™ III First‐Strand Synthesis SuperMix (18080400), with specific primers for miR‐21, miR‐146a, and internal controls GAPDH and U6 snRNA available with the kit. The reaction mixture (20 μL) was incubated at 42°C for 1 h and terminated at 85 °C for 5 min.

Quantitative PCR was performed using SYBR Green dye. Reaction mixtures (10 μL) contained 1 ng cDNA, primers, and a master mix, with amplification carried out on a magnetic induction real‐time PCR cycler. The delta–delta Ct (ΔΔCt) method [26] was used to calculate relative expression levels of *INSR*, miR‐21, and miR‐146a, normalized to internal controls (Table 2).

**TABLE 2:** Primer sequences and annealing temperatures of microRNAs for real‐time PCR.

Gene/microRNA	Primer	Primer sequences	Annealing temperature
miR‐21	Forward	5′ TAGCTTATCAGACTGATGTTGA 3′	45
	Reverse	5′ GTGCAGGGTCCGAGGT 3′	

miR‐146a	Forward	5′ TGAGAACTGAATTCCATGGGTT 3′	48.5
	Reverse	5′ GTGCAGGGTCCGAGGT 3′	

Clinical, biochemical, and demographic data were analyzed using SPSS Version 22 and GraphPad Prism Version 8. Comparisons between groups were made using *t*‐tests, ANOVA, and nonparametric Mann–Whitney *U* tests. The diagnostic value of miRNAs was evaluated using the receiver‐operating characteristic (ROC) curves and area‐under‐the‐curve (AUC) metrics [27]. Gene expression differences were assessed using REST software and Pfaffl’s method. Statistical significance was set at *p* < 0.05.

## 3. Results

Comparison of biochemical, clinical, and demographic characteristics of Type 2 diabetics (T2DM) with the controls exhibited significantly elevated FPG, HbA1c, cholesterol levels, blood pressure, and BMI in patients (*p* < 0.05). Significant differences were also observed for age and weight (*p* < 0.05). For males, occupational stress, high BMI, and advancing age exhibited a stronger association with T2DM risk. Conversely, among females, lack of exercise, obesity, and mental stress were the predominant factors contributing to disease development (Table 3) (Supporting 4).

**TABLE 3:** Biochemical, clinical, and demographic profile of respondents (T2DM patients and healthy subjects).

Variables	Diseased respondents	Healthy respondents	Significance (*p* value)
Total respondents	*n* = 400	*n *= 300	——
Gender %			——
Male	62.5	60	——
Female	37.5.	40	
Age (year), mean, ±SD	49.19 ± 18.27	46.38 ± 14.63	0.028^∗^
Height (cm), mean, ±SD	169.12 ± 4.02	171.03 ± 5.71	< 0.0001^∗^
Weight (kg), mean, ±SD	65.25 ± 11.56	67.10 ± 13.05	0.047^∗^
BMI (kg/m^2^), mean, ±SD	25.52 ± 3.91	27.24 ± 4.10	< 0.0001
FPG (mmol/L)			
Fasting plasma glucose	8.71 ± 0.79	7.44 ± 1.10	< 0.0001
HbA1c (%)	10.12 ± 1.97	4.43 ± 1.00	< 0.0001
Glycated hemoglobin A12			
HDL cholesterol (mmol/L)	1.12 ± 0.32	1.03 ± 1.10	0.012
LDL cholesterol (mmol/L)	3.96 ± 0.52	2.6 ± 0.5	0.0002
Systolic BP (mmHg)	138.37 ± 5.47	118.01 ± 4.57	< 0.0001
Diastolic BP (mmHg)	88.47 ± 6.98	76.24 ± 4.47	< 0.0001

^∗^Mann–Whitney U test (W statistics) for comparison between variables of two groups.

Using Sanger sequencing, three SNPs, rs1799817 (C > T) located at Position 19:7112553 in the reverse strand; rs1052371, a deletion mutation identified in the forward strand; and rs1799816 (G> A) in the forward strand at Position 19:7112582 (Supporting 5a, 5b, and 5c), were found. All these SNPs (Supporting 6) were found to be contributing factors to T2DM susceptibility (*p* < 0.05) in the studied population by using a comprehensive analysis (GENCODE 44) tool.

Three novel mutations were also detected through sequencing: a forward strand deletion mutation at Codon 186 (GAT ⟶ GA‐), with no significant association with T2DM; reverse strand base substitution (CAC ⟶ CAT, His1058His) at Codon 115; and forward strand nucleotide substitution (GGT ⟶ GAT) at Codon 83. *In silico* analysis using tools such as PolyPhen‐2 and SIFT predicted the potential impact of novel and rare variants on protein structure and function (Supporting 7). The results indicate that the substitution mutations at Codons 83 and 115 are likely benign/tolerated, while the deletion at Codon 186 may affect local structure but showed no disease association in our dataset.

**FIGURE 1:**
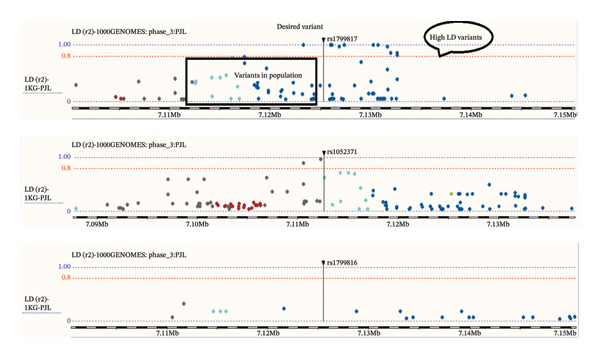
Linkage disequilibrium *r* ^2^ score. On y axis, LD *r* ^2^ score 0.8–1.00 shows the score of desired variants as well as nearby variants in a selected population. Peak identifies (A) rs1799817 B, rs1052371, and (C) rs1799816 LD r^2^ score and SNP association with disease.

Linkage disequilibrium (LD) (Figure 1) indicated the physical positions and associations of the variants within the population. SNPs with LD scores (*r* ^2^ > 0.8) demonstrated strong linkage to T2DM risk.

*INSR* gene expression was significantly downregulated in T2DM patients, with a 92.5% reduction in mRNA levels compared to controls (SD = 0.53; SE = 0.30 for controls and SD = 0.05; SE = 0.03 for T2DM).

**FIGURE 2:**
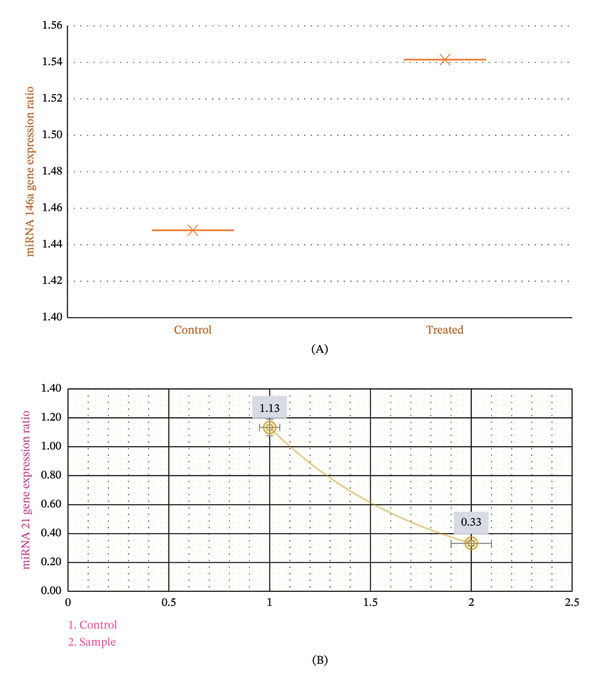
Expression ratio of differentially expressed miRNAs in T2D patients and controls. (A) Increase of miRNA‐146a expression in patients. (B) miRNA‐21 ratio decreased in diabetic group. Control group SEM (0.34) vs. SD (0.58) and sample group SEM (0.16) vs. SD (0.27).

Quantitative PCR results highlighted differential expression of circulating miRNAs in T2DM with miRNA‐146a upregulation in 6.2% of T2DM patients and miRNA‐21 downregulation in 70.79% of controls (Figure 2).

**FIGURE 3:**
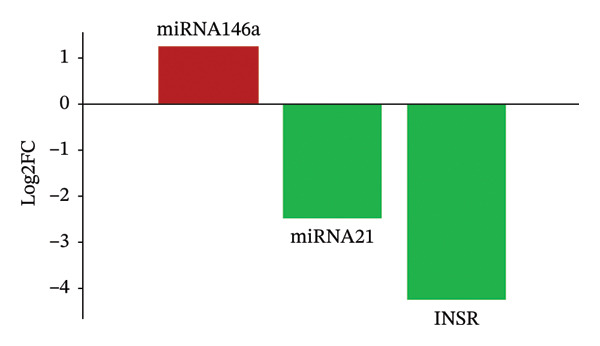
Relative quantification plot. It determines upregulation of miRNA‐146a, downregulation of miRNA‐21 and *INSR* mRNA relative to the reference (exogenous and endogenous) sample.

Log2 fold change analysis (Figure 3) confirmed the downregulation of the *INSR* gene in T2DM patients, strongly linked to disease risk (*p* < 0.05), and miRNA‐146a upregulation and negative fold change for miRNA‐21, signifying downregulation.

### 3.1. Correlation Analysis

Pearson correlation analysis (Supporting 8) revealed positive correlations for *INSR* with FPG, HbA1c, and LDL (*p* < 0.001), and miRNA‐21 was negatively correlated with FPG but positively with HbA1c and LDL. miRNA‐146a showed positive correlations with FPG and HbA1c but negative with LDL. No significant correlations were observed between genes/miRNAs and blood pressure parameters.

ROC analysis demonstrated the diagnostic potential of circulating miRNAs (Supporting 9 and 10) (miRNA‐21: AUC = 0.792; sensitivity = 80.7%; specificity = 65.2%) (95% CI: 0.66–0.94; *p* = 0.0043) (miRNA‐146a: AUC = 0.755; sensitivity = 90%; specificity = 65%) (95% CI: 0.63–0.92; *p* = 0.001). These findings indicate the utility of combined miRNA‐21 and miRNA‐146a detection as reliable biomarkers for T2DM.

## 4. Discussion

A significant association of SNPs located in Exon 17 of the *INSR* gene with the prognosis of T2DM was identified [28, 29]. The forward and reverse strands of the *INSR* gene were sequenced, revealing key insights into genetic variations. Notably, the previously reported polymorphism rs1799817 (C > T), resulting in a synonymous substitution His1085His, was detected. This SNP is consistent with findings from prior studies [30], such as Bodhini et al. [31], who identified this variant using PCR‐RFLP, and Conway et al. [32], where eight patients were reported to carry the C/T substitution [33, 34]. In contrast, rs1052371 demonstrated a deletion mutation in the current study, which did not provide evidence of a direct role in T2DM development. Interestingly, we identified a novel mutation, rs1799816 (G> A), in the sequenced data. This discovery adds to the growing understanding of genetic variations influencing T2DM risk in diverse populations. Few studies have explored the relationship of *INSR* Exon 17 polymorphisms with T2DM [35–40]. For instance, Bodhini et al. [31] confirmed the association of the His1085His polymorphism with T2DM in an Indian population. Similarly, Conway et al. [32] observed the C/T substitution mutation in eight individuals, underscoring the relevance of this SNP in various populations. However, the discovery of the novel rs1799816 mutation in the present study emphasizes the need for further research to explore its functional implications and its role in T2DM development [41].

Quantitative analysis of *INSR* gene expression and circulating miRNAs (miRNA‐21 and miRNA‐146a) further highlighted their roles in T2DM pathogenesis. *INSR* mRNA expression was significantly downregulated, whereas miRNA‐21 exhibited marked downregulation (70.79% reduction) [12, 42–44] and miRNA‐146a showed upregulation (6.2% increase) in T2DM patients compared to controls [19, 45–48]. These changes in gene and miRNA expression were strongly correlated with clinical and biochemical markers, particularly FPG, HbA1c, and low‐density lipoprotein (LDL) levels [49]. These factors appear to play a pivotal role in modulating gene and miRNA expression, influencing the progression of diabetes [50].

This study indicates the intricate interplay of genetic variations and molecular markers in the etiology of T2DM, providing valuable insights into potential biomarkers and therapeutic targets for diabetes management in the Pakistani population.

## 5. Conclusion

This study contributes additional evidence within the Pakistani population demonstrating the possible susceptible association of SNPs with the expression profile of the *INSR* gene and circulatory miRNA‐21 and miRNA‐146A in T2DM patients. Since a limited number of candidate variants were analyzed, formal multiple testing correction was not applied. Nevertheless, we acknowledge that multiple comparisons may increase the likelihood of false‐positive results, and therefore, the findings should be interpreted cautiously. Cross‐sectional design, independent validation cohort, functional studies of identified SNPs, larger population‐based validation, and longitudinal tracking of miRNA expression dynamics need to be done in future.

## Funding

No funding was received for this study.

## Conflicts of Interest

The authors declare no conflicts of interest.

## Supporting Information

Additional supporting information can be found online in the Supporting Information section.

## Supporting information


**Supporting Information** Supporting 1. Ideogramic view of Chromosome 19. The red square shows the position of the INSR gene. Supporting 2. INSR genomic sequence location on Chromosome 19:7112202:7112991. The minus (−) sign on top of the figure shows the reverse orientation of the sequence. The marked nucleotide shows the original position of the base in the INSR gene. Supporting 3. Forward orientation of the INSR genomic sequence. The position number of nucleotides in human genome is highlighted in yellow color. Supporting 4. Fraction of the total contingency. Mean, standard deviation, and strength of association between risk factors, group, and prognosis of T2DM in the present study. Bars indicate the mean, and error bars and dots show the probability of T2DM. Supporting 5a. Alignment results of rs1799817 C > T. Total length of query was 190 bp. The highlighted region shows that C is replaced by T at Position no. 115. A mutation was found on the reverse strand of the INSR gene. Supporting 5b. Pairwise alignment picture of rs1052371. A deletion mutation was found in the forward strand of the INSR gene. Supporting 5c. In the forward strand of the INSR gene, Nucleotide G is replaced with A, thus confirming the rs1799816 polymorphism in T2 diabetic patients. Supporting 6. Nucleotide sequencing of Exon 17 of the INSR gene. (A) Single nucleotide deletion mutation. (B and C) Base substitution mutation in T2 diabetic patients of Pakistani population. Supporting 7: Functional effects of detected variants were predicted using PolyPhen‐2 and SIFT. PolyPhen‐2 predicts the possible impact of amino acid substitutions on protein structure and function, whereas SIFT evaluates whether substitutions are tolerated based on sequence homology and physicochemical properties of amino acids. Supporting 8. Correlation of INSR, miRNA‐21, and miRNA‐146a expression with clinical parameters. Supporting 9. Box plot with Jetter and *p* values. High risk of T2DM is associated with downregulation of INSR gene expression. *p* < 0.05 (Wilcox test) shows statistical significance. Supporting 10. ROCs curve for capacity of miRNA‐21 and miRNA‐146a to compute the diagnostic values for T2DM. Sensitivity is the total number of people who have tested positive for the target disease (T2DM), and specificity reflects control (tested negative) of study. AUC measures the test’s accuracy (AUC > 0.7). The closer the ROC curve to the upper left corner of the graph (near Sensitivity 1), the higher will be the test’s accuracy.

## Data Availability

The data that support the findings of this study are available in the Supporting Information of this article.
