# Supplementary material for: Genomic and Transcriptomic Variations of INSR Are Associated With Dysregulation of Circulatory miRNA‐21 and miRNA‐146a in Type 2 Diabetes Mellitus
Source: J Nutr Metab. 2026 Jul 22;2026:9980111. doi: 10.1155/jnme/9980111 (PMC13392512; doi:10.1155/jnme/9980111)
Supplement: Supplementary file 1 — Supporting Information Supporting 1. Ideogramic view of Chromosome 19. The red square shows the position of the INSR gene. Supporting 2. INSR genomic sequence location on Chromosome 19:7112202:7112991. The minus (−) sign on top of the figure shows the reverse orientation of the sequence. The marked nucleotide shows the original position of the base in the INSR gene. Supporting 3. Forward orientation of the INSR genomic sequence. The position number of nucleotides in human genome is highlighted in yellow color. Supporting 4. Fraction of the total contingency. Mean, standard deviation, and strength of association between risk factors, group, and prognosis of T2DM in the present study. Bars indicate the mean, and error bars and dots show the probability of T2DM. Supporting 5a. Alignment results of rs1799817 C > T. Total length of query was 190 bp. The highlighted region shows that C is replaced by T at Position no. 115. A mutation was found on the reverse strand of the INSR gene. Supporting 5b. Pairwise alignment picture of rs1052371. A deletion mutation was found in the forward strand of the INSR gene. Supporting 5c. In the forward strand of the INSR gene, Nucleotide G is replaced with A, thus confirming the rs1799816 polymorphism in T2 diabetic patients. Supporting 6. Nucleotide sequencing of Exon 17 of the INSR gene. (A) Single nucleotide deletion mutation. (B and C) Base substitution mutation in T2 diabetic patients of Pakistani population. Supporting 7: Functional effects of detected variants were predicted using PolyPhen‐2 and SIFT. PolyPhen‐2 predicts the possible impact of amino acid substitutions on protein structure and function, whereas SIFT evaluates whether substitutions are tolerated based on sequence homology and physicochemical properties of amino acids. Supporting 8. Correlation of INSR, miRNA‐21, and miRNA‐146a expression with clinical parameters. Supporting 9. Box plot with Jetter and p values. High risk of T2DM is associated with downregulation of I [file JNME-2026-9980111-s001.docx]

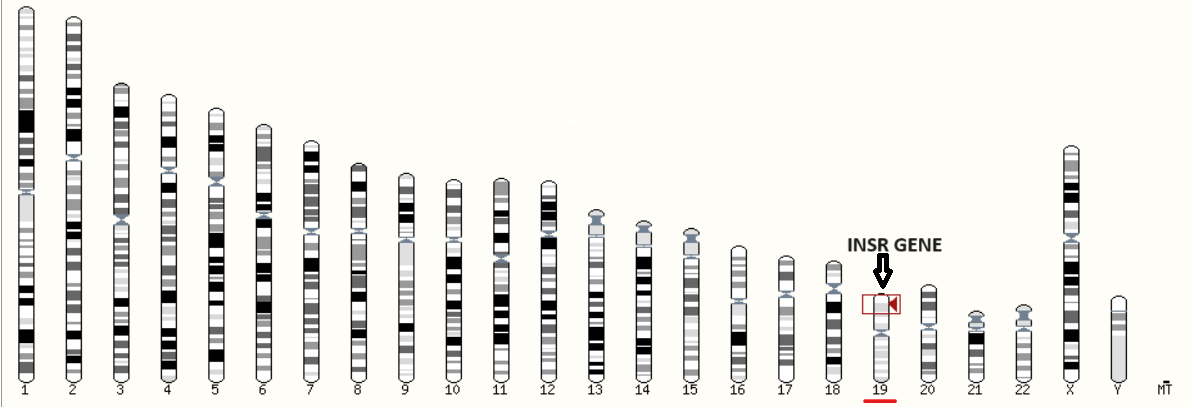


Supplementary 1. Ideogramic view of chromosome 19. The red square shows the position of the INSR gene.

**
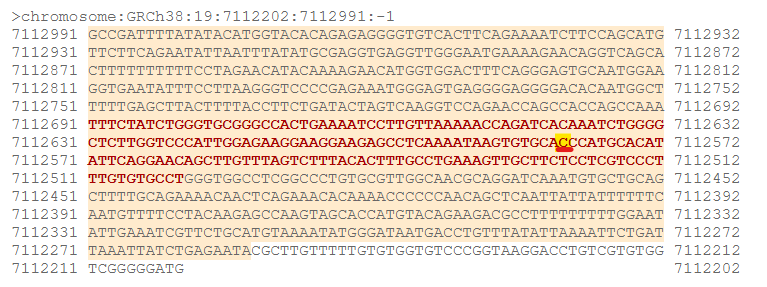
**

Supplementary 2. INSR genomic sequence location on chromosome 19:7112202:7112991. The Minus (-) sign on top of the figure shows the reverse orientation of the sequence. Marked nucleotide shows the original position of the base in the INSR gene.


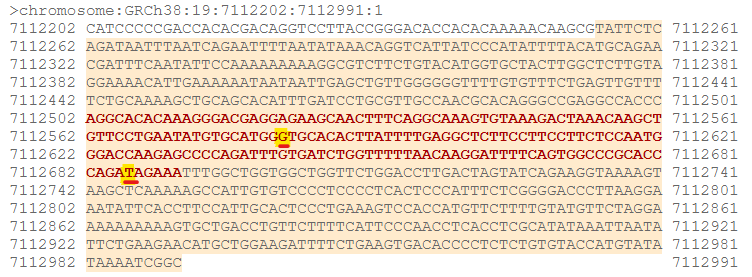


Supplementary 3. Forward orientation of INSR genomic sequence. The position no of nucleotides in human genome is highlighted in yellow color.


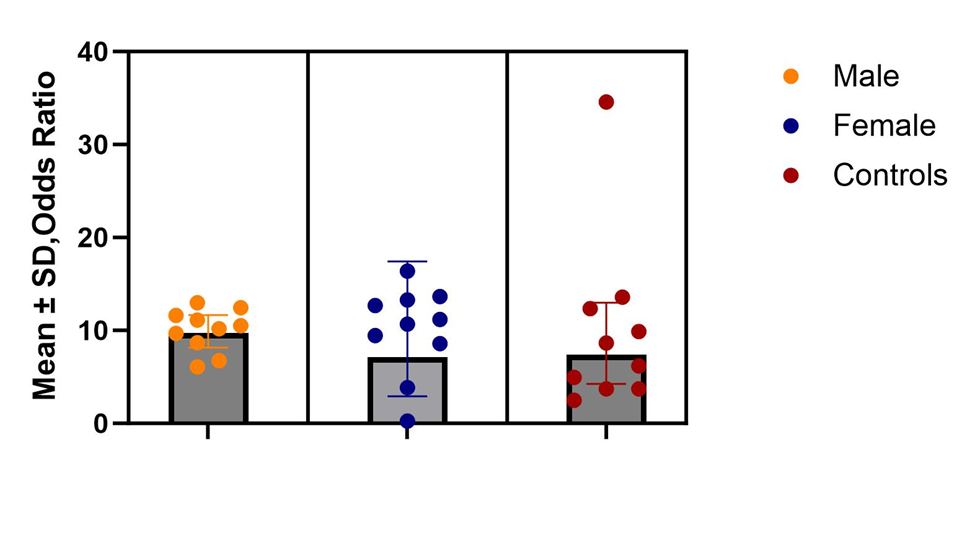


Supplementary 4. Fraction of total of contingency. Mean, standard deviation, and strength of association between risk factors, group, and prognosis of T2DM in the present study. Bars indicate the mean and error bars and dots show the probability of T2DM.

**
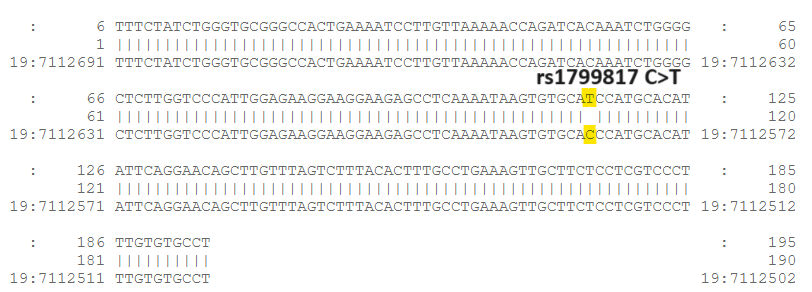
**

Supplementary 5a. Alignment results of rs1799817 C>T. Total length of query was 190bp. Highlighted region shows that C is replaced by T at position no 115. Mutation was found at the reverse strand of the INSR gene.

**
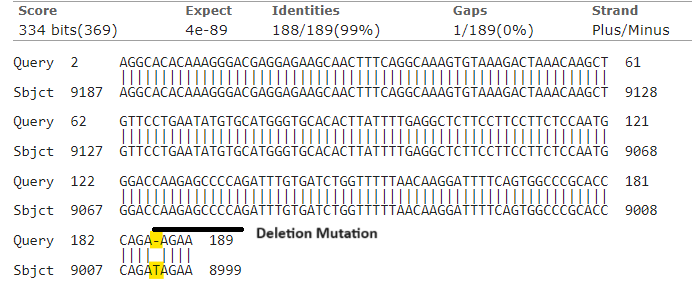
**

Supplementary 5b. Pairwise alignment picture of rs1052371 T>C. Deletion mutation was found in forward strand of INSR gene.

**
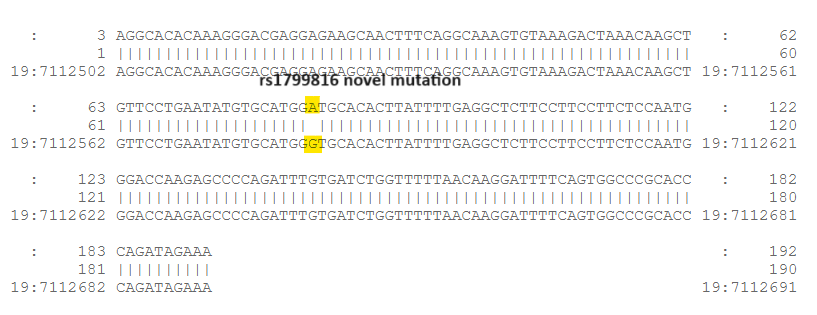
**

Supplementary 5c. In the forward strand of INSR gene nucleotide G is replaced with A hence confirming the rs1799816 polymorphism in T2 diabetic patients.


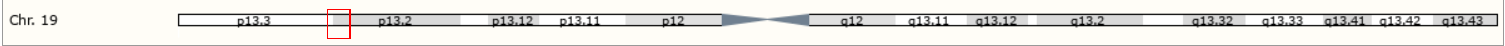


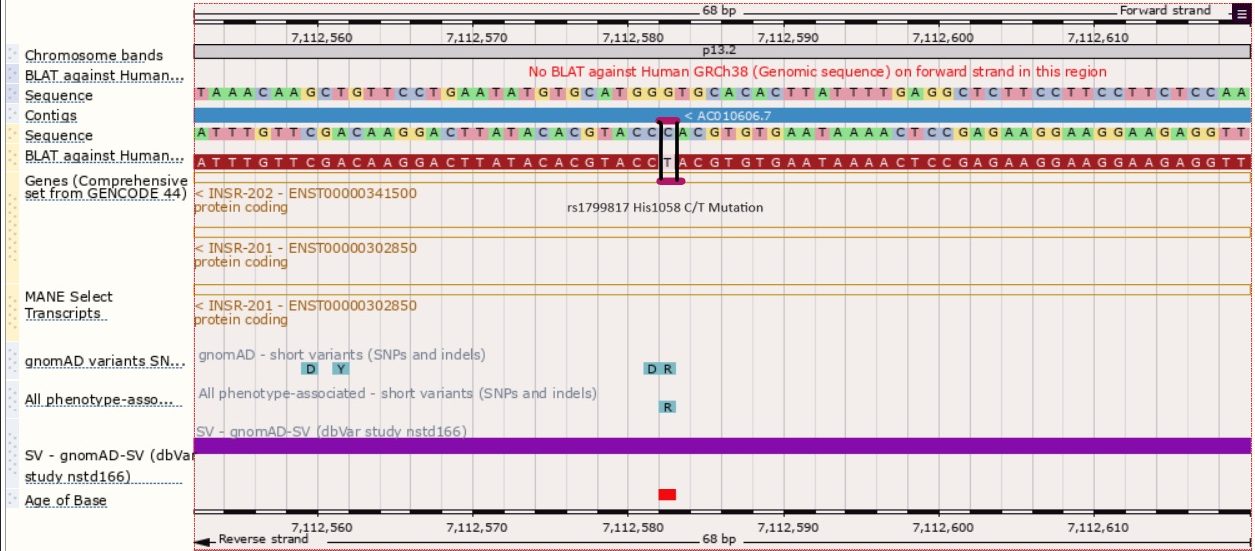


**
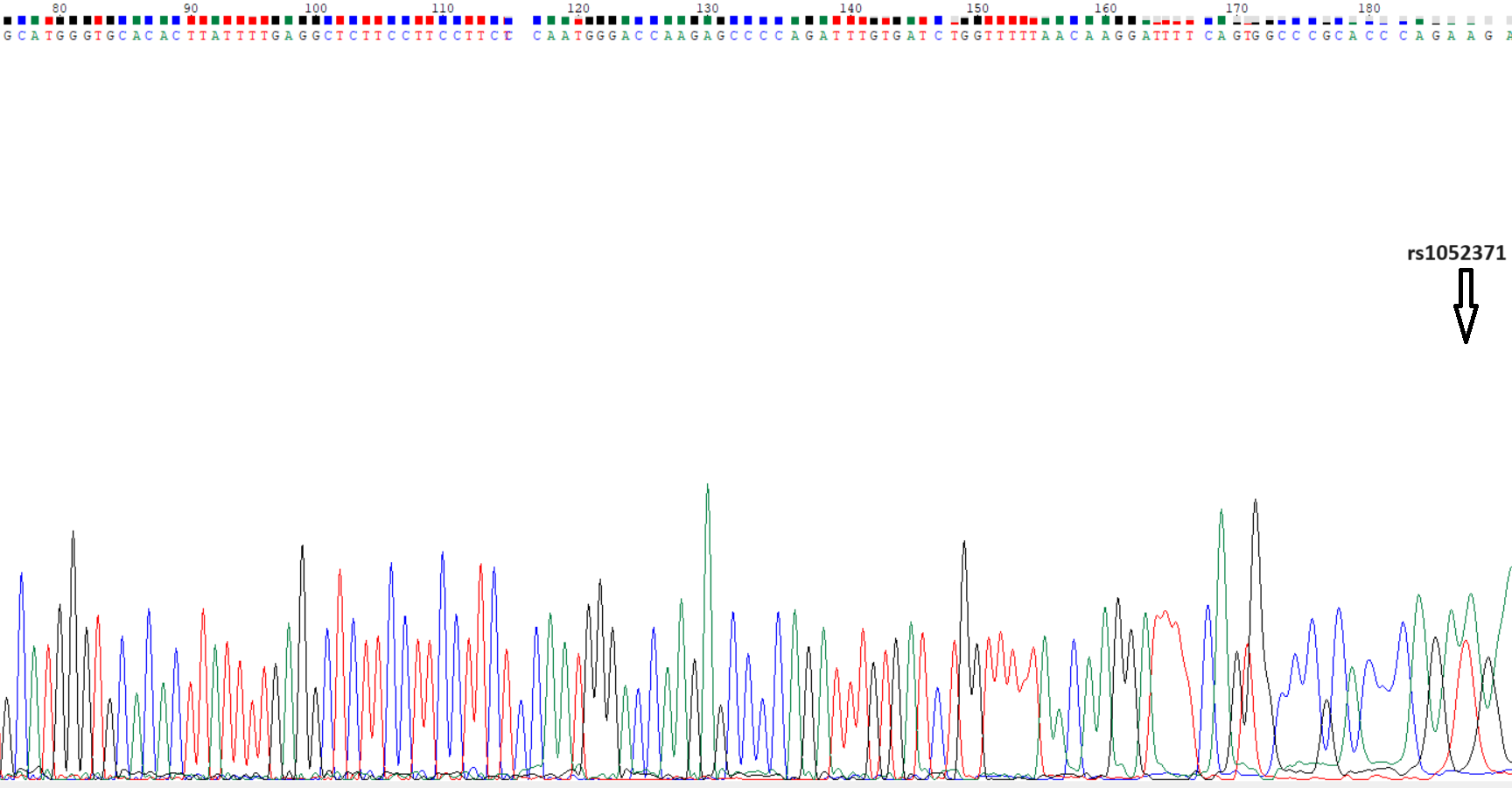
**


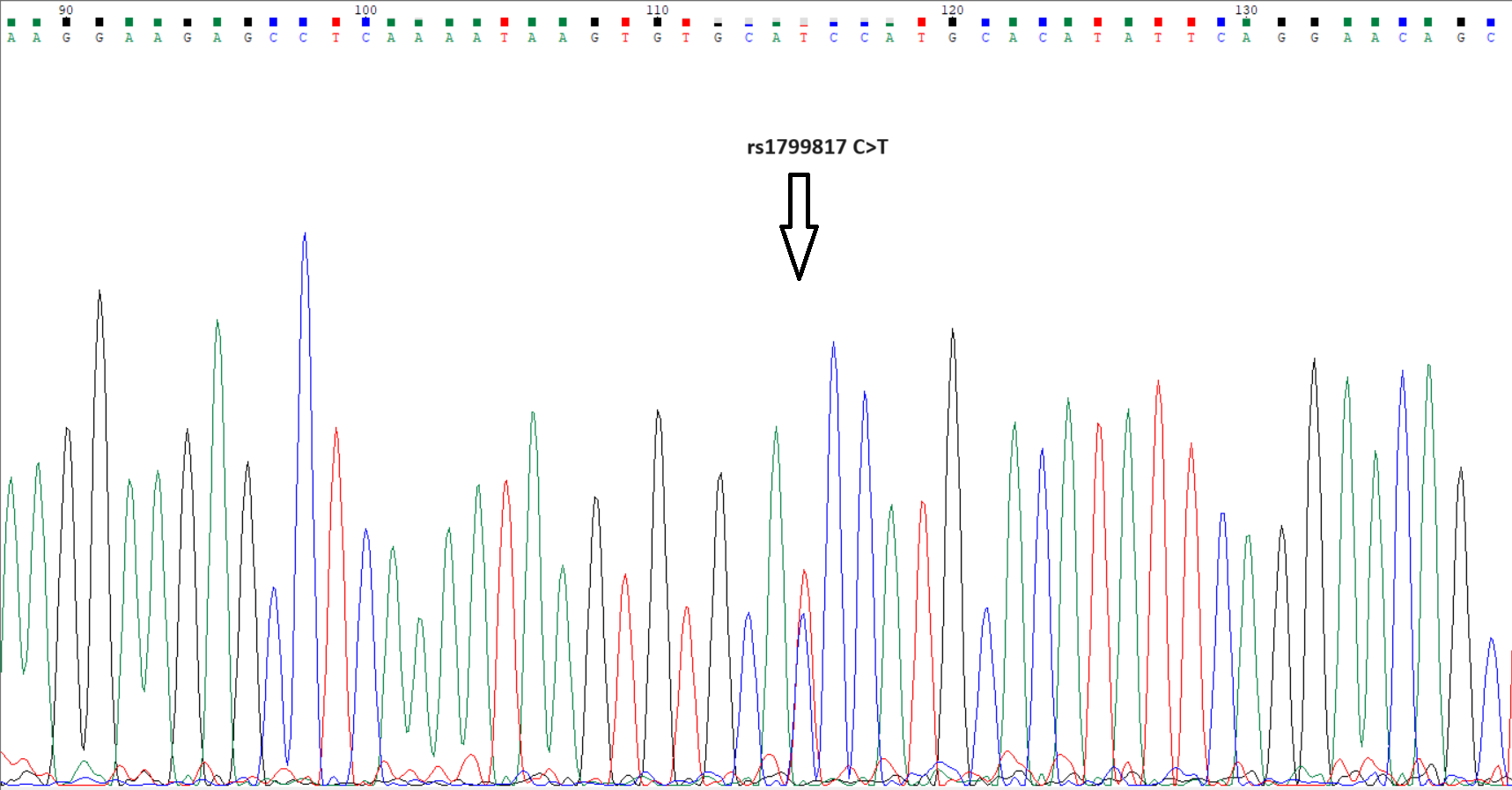


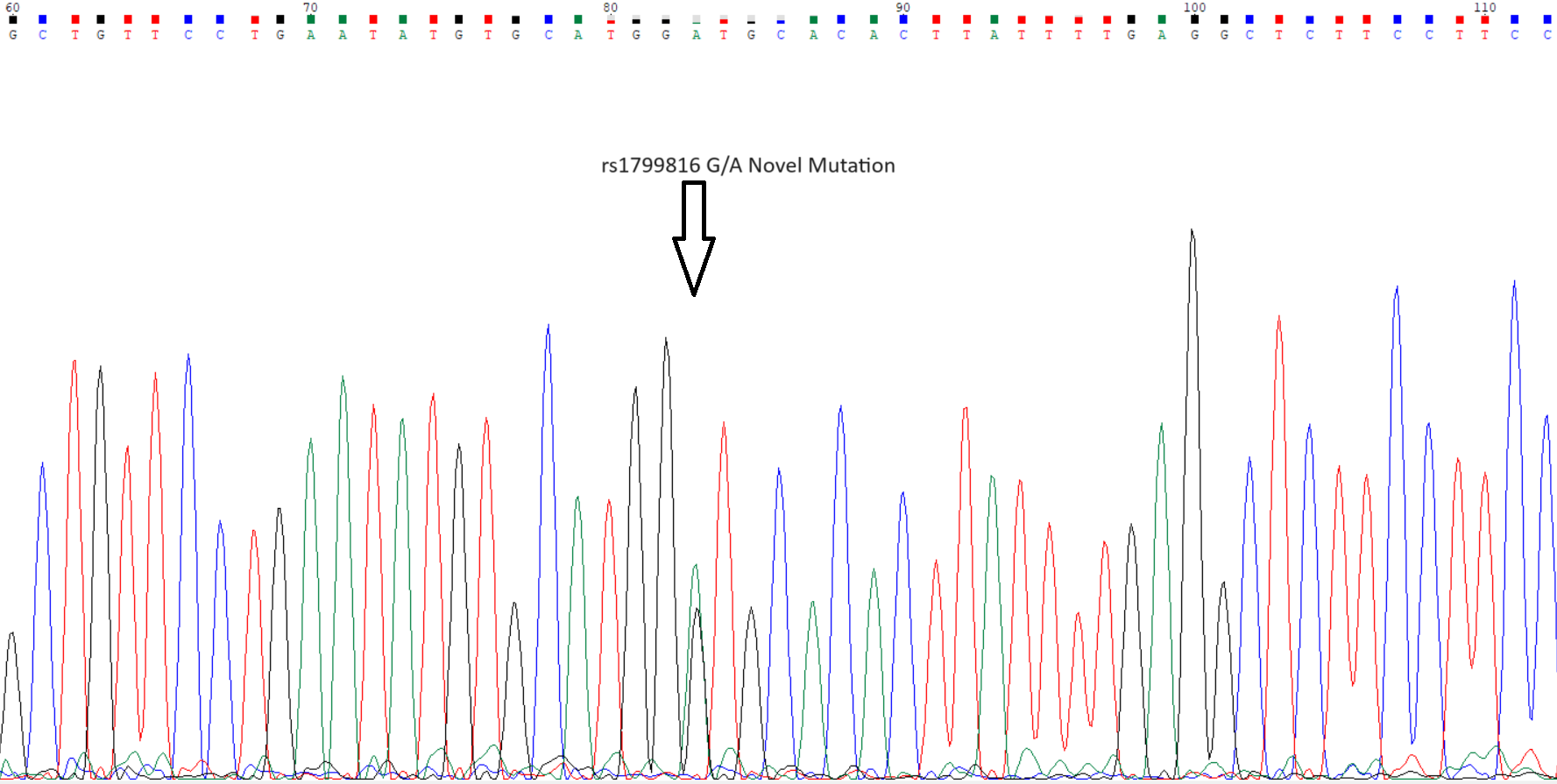


Supplementary 6. Nucleotide sequencing of exon 17 of INSR gene. A, Single nucleotide deletion mutation. b & c, Base substitution mutation in T2 diabetic patients of Pakistani population.

Supplementary 7: Functional effects of detected variants were predicted using PolyPhen-2 and SIFT. PolyPhen-2 predicts the possible impact of amino acid substitutions on protein structure and function, whereas SIFT evaluates whether substitutions are tolerated based on sequence homology and physicochemical properties of amino acids.

| **Codon Position** | **Strand** | **Nucleotide Change** | **Amino Acid Change** | **Mutation Type** | **PolyPhen-2 Prediction** | **SIFT Prediction** | **Functional Interpretation** |
| --- | --- | --- | --- | --- | --- | --- | --- |
| 186 | Forward | GAT → GA- | Asp186del | Deletion | Possibly damaging | Not applicable | May affect local protein structure but showed no association with T2DM |
| 115 | Reverse | CAC → CAT | His1058His | Synonymous substitution | Benign | Tolerated | No predicted functional impact |
| 83 | Forward | GGT → GAT | Gly83Asp | Missense substitution | Benign | Tolerated | Likely neutral variant |

Supplementary 8. Correlation of INSR, miRNA21, miRNA146a expression with clinical parameters

| \| Indicators \| FPG HBA1c HDL \| LDL SBP DBP \|  \| \| --- \| --- \| --- \| --- \| \|  \|  \|  \|  \|   INSR  r 0.465 0.421 0.184 0.158^a^ -0.052 0.139  *p* <0.001 <0.001 0.014 <0.001 0.458 0.060  miRNA 21  r -0.018 0.499 -0.376 0.201^a^ 0.062 0.074  *p* 0.635 <0.001 0.063 <0.001 0.186 0.230  miRNA146a  r 0.187 0.443 -0.217 0.136^a^ -0.651 -0.437  *p*  <0.001 <0.001 0.080 0.653 0.179 0.772 |
| --- | --- | --- | --- | --- | --- | --- | --- | --- |

Note: Data was analyzed using log of 2^-deltaCT^ of INSR, miRNA21 and miRNA146a along with variables by Pearson correlation and ^a^Spearman correlation test.

Abbreviations: FPG (fasting plasma glucose), HbA1c (glycated hemoglobin), HDL (high density lipoprotein), LDL (low density lipoprotein), SBP (systolic blood pressure) and DBP (diastolic blood pressure).


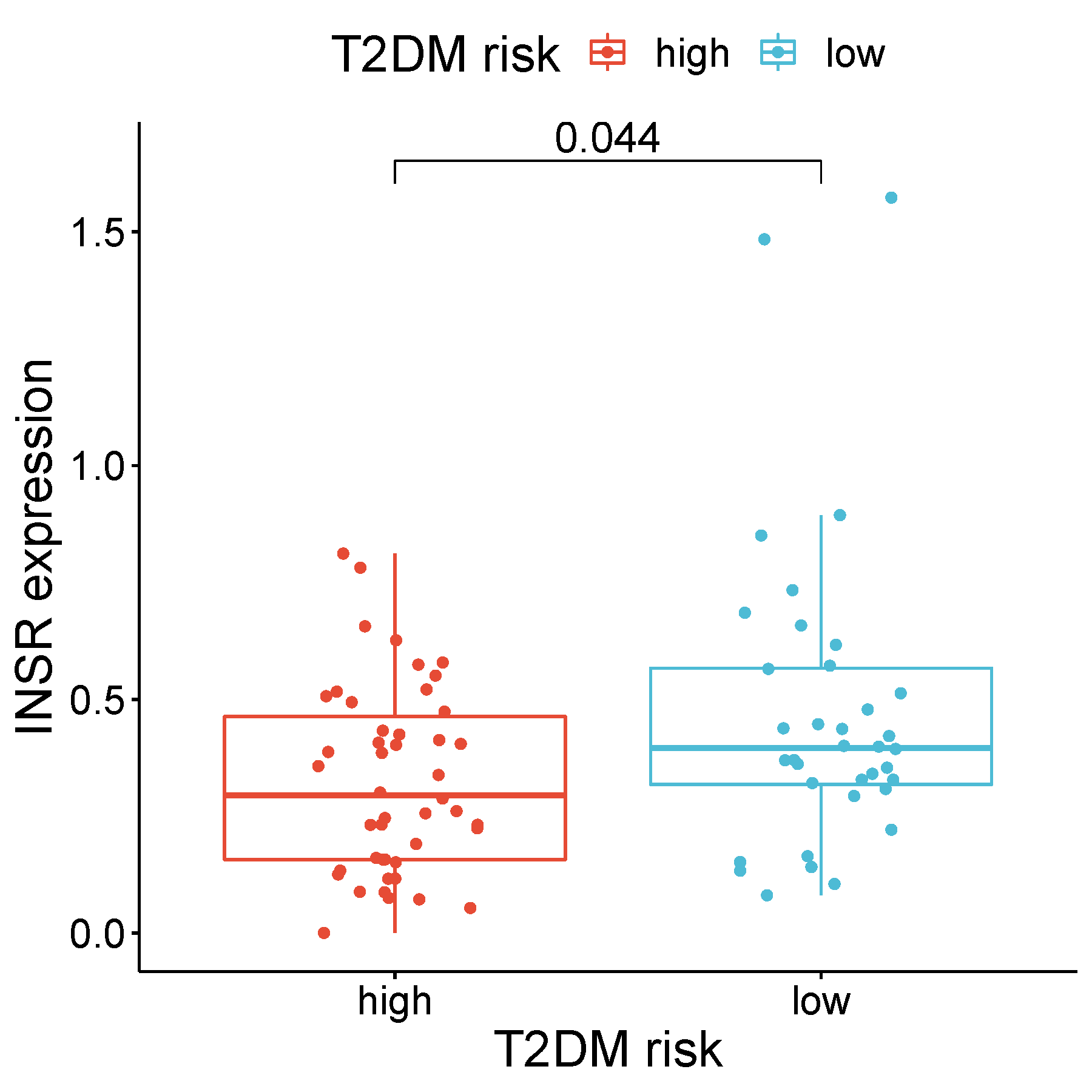


Supplementary 9. Box plot with Jetter and p-values. High risk of T2DM is associated with down regulation of INSR gene expression. *P<0.05* (Wilcox test) shows statistical significance.


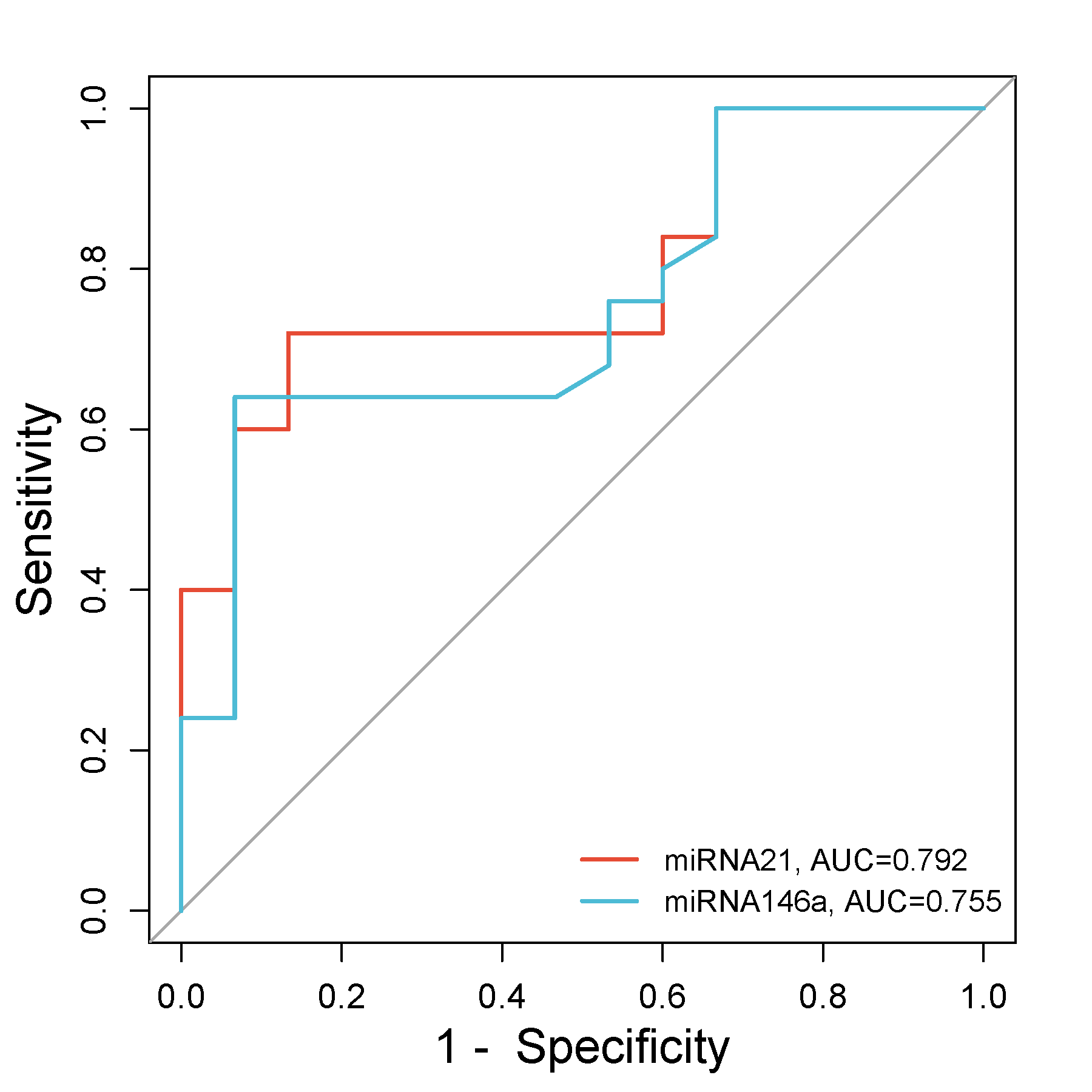


Supplementary 10. Receiver operating characteristics (ROC) curve for capacity of miRNA21 and miRNA146a to compute the diagnostic values for T2DM. Sensitivity is the total number of people who have tested positive for target disease (T2DM), and specificity reflects control (tested negative) of study. AUC measures the test’s accuracy (AUC > 0.7). The closer ROC cure to upper left corner of graph (near sensitivity 1), the higher will be the test’s accuracy.
